# Supplementary material for: Machine Learning Accelerates Crystallization for Structure Determination
Source: Angew Chem Int Ed Engl. 2026 May 4;65(25):e1218503. doi: 10.1002/anie.1218503 (PMC13266958; doi:10.1002/anie.1218503)

checkCIF/PLATON report

Structure factors have been supplied for datablock(s) lcz024

THIS REPORT IS FOR GUIDANCE ONLY. IF USED AS PART OF A REVIEW PROCEDURE FOR PUBLICATION, IT SHOULD NOT REPLACE THE EXPERTISE OF AN EXPERIENCED CRYSTALLOGRAPHIC REFEREE.

No syntax errors found. CIF dictionary Interpreting this report

Datablock: lcz024

Bond precision:	C-C = 0.0072 A		Wavelength=1.54184
Cell:	a=13.4249(2)	b=13.2053(1)	c=17.2600(2)
	alpha=90	beta=90.353(1)	gamma=90
Temperature:	100 K		

	Calculated	Reported
Volume	3059.79(6)	3059.79(6)
Space group	P 21/c	P 1 21/c 1
Hall group	-P 2ybc	-P 2ybc
Moiety formula	C24 H11 Ag3 F18 N6 O2	C15 H3 Ag3 F18 N6, C9 H8 O2
Sum formula	C24 H11 Ag3 F18 N6 O2	C24 H11 Ag3 F18 N6 O2
Mr	1081.00	1081.00
Dx,g cm-3	2.347	2.347
Z	4	4
Mu (mm-1)	16.651	16.651
F000	2064.0	2064.0
F000'	2074.18	
h,k,lmax	17,16,21	16,16,21
Nref	6538	6141
Tmin,Tmax	0.112,0.097	0.574,1.000
Tmin'	0.031	

Correction method= # Reported T Limits: Tmin=0.574 Tmax=1.000
AbsCorr = MULTI-SCAN

```
Data completeness= 0.939            Theta(max)= 78.051

                                                        wR2(reflections)=
R(reflections)= 0.0423( 5477)                           0.1172( 6141)

S = 1.073                Npar= 478
```

The following ALERTS were generated. Each ALERT has the format
test-name_ALERT_alert-type_alert-level.
Click on the hyperlinks for more details of the test.

Alert level C

```
PLAT029_ALERT_3_C _diffrn_measured_fraction_theta_full value Low .      0.975 Why?
PLAT042_ALERT_1_C Calc. and Reported MoietyFormula Strings  Differ     Please Check
              Calc: C24 H11 Ag3 F18 N6 O2
              Rep.: C15 H3 Ag3 F18 N6, C9 H8 O2
PLAT767_ALERT_4_C INS Embedded LIST 6 Instruction Should be LIST 4     Please Check
PLAT906_ALERT_3_C Large K Value in the Analysis of Variance ......      2.098 Check
PLAT911_ALERT_3_C Missing FCF Refl Between Thmin & STh/L=    0.600        140 Report
                1  1  0,   6 13  1,   6 14  1,   0  0  2,   1  0  2,  -6  3  2,
                1  8  2,   5 13  2,   5 14  2,   6 14  2,   1  8  3,   4 13  3,
               -2  0  4,   8  0  4,  -7  2  5,  10  0  6,  11  0  6,   5  5  6,
                1 15  6, -14  0  8,  -6  0  8,  14  1  8,  14  2  8,   9  8  8,
                9  9  8,   9 10  8,  -3  2  9,   6  7  9,   9  9  9,  10  9  9,
                   (  110 More Missing: see the .ckf listing file)
```

Alert level G

```
PLAT142_ALERT_4_G s.u. on b - Axis Small or Missing .............    0.00010 Ang.
PLAT242_ALERT_2_G Low    'MainMol' Ueq as Compared to Neighbors of       C00X Check
PLAT242_ALERT_2_G Low    'MainMol' Ueq as Compared to Neighbors of       C00Y Check
PLAT242_ALERT_2_G Low    'MainMol' Ueq as Compared to Neighbors of       C019 Check
PLAT242_ALERT_2_G Low    'MainMol' Ueq as Compared to Neighbors of       C01B Check
PLAT242_ALERT_2_G Low    'MainMol' Ueq as Compared to Neighbors of       C01D Check
PLAT242_ALERT_2_G Low    'MainMol' Ueq as Compared to Neighbors of       C01E Check
PLAT434_ALERT_2_G Short Inter HL..HL Contact F005     ..F00D     .       2.79 Ang.
                                                  x,1+y,z  =      1_565 Check
PLAT434_ALERT_2_G Short Inter HL..HL Contact F007     ..F008     .       2.82 Ang.
                                          1-x,1/2+y,1/2-z  =      2_655 Check
PLAT720_ALERT_4_G Number of Unusual/Non-Standard Labels ..........         64 Note
              Ag01    Ag02    Ag03    F004    F005    F006    F007    F008
              F009    F00A    F00B    F00C    F00D    F00E    F00F    N00G
              F00H    O00I    F00J    O00K    F00L    F00M    N00N    F00O
              N00P    N00Q    N00R    N00S    F00T    C00U    H00U    C00V
              C00W    H00A    H00B    C00X    C00Y    C00Z    C010    C011
              C012    H012    C013    C014    C015    C016    H016    C017
              C018    H01A    H01B    C019    C01A    C01B    C01C    H01C
              C01D    C01E    C01F    H01F    C01G    H01G    C01H    H01H
PLAT912_ALERT_4_G Missing # of FCF Reflections Above STh/L=  0.600        255 Note
PLAT913_ALERT_3_G Missing # of Very Strong Reflections in FCF ....          1 Note
               -2  0  4,
PLAT941_ALERT_3_G Average HKL Measurement Multiplicity ..........        3.0 Low
PLAT969_ALERT_5_G The 'Henn et al.' R-Factor-gap value ..........      2.796 Note
              Predicted wR2: Based on SigI**2  4.19 or SHELX Weight 10.93
```

```
PLAT978_ALERT_2_G Number C-C Bonds with Positive Residual Density.          0 Info
```

```
   0 ALERT level A = Most likely a serious problem - resolve or explain
   0 ALERT level B = A potentially serious problem, consider carefully
   5 ALERT level C = Check. Ensure it is not caused by an omission or oversight
  15 ALERT level G = General information/check it is not something unexpected

   1 ALERT type 1 CIF construction/syntax error, inconsistent or missing data
   9 ALERT type 2 Indicator that the structure model may be wrong or deficient
   5 ALERT type 3 Indicator that the structure quality may be low
   4 ALERT type 4 Improvement, methodology, query or suggestion
   1 ALERT type 5 Informative message, check
```

Validation response form

Please find below a validation response form (VRF) that can be filled in and pasted into your CIF.

```
# start Validation Reply Form
_vrf_PLAT029_lcz024
;
PROBLEM: _diffrn_measured_fraction_theta_full value Low .      0.975 Why?
RESPONSE: ...
;
_vrf_PLAT042_lcz024
;
PROBLEM: Calc. and Reported MoietyFormula Strings  Differ     Please Check
RESPONSE: ...
;
_vrf_PLAT767_lcz024
;
PROBLEM: INS Embedded LIST 6 Instruction Should be LIST 4     Please Check
RESPONSE: ...
;
_vrf_PLAT906_lcz024
;
PROBLEM: Large K Value in the Analysis of Variance ......      2.098 Check
RESPONSE: ...
;
_vrf_PLAT911_lcz024
;
PROBLEM: Missing FCF Refl Between Thmin & STh/L=    0.600        140 Report
RESPONSE: ...
;
# end Validation Reply Form
```

It is advisable to attempt to resolve as many as possible of the alerts in all categories. Often the minor alerts point to easily fixed oversights, errors and omissions in your CIF or refinement strategy, so attention to these fine details can be worthwhile. It is up to the individual to critically assess their own results and, if necessary, seek expert advice.

PLATON version of 17/09/2025; check.def file version of 17/09/2025

duplicate check

No duplication found

Datablock lcz024 - ellipsoid plot

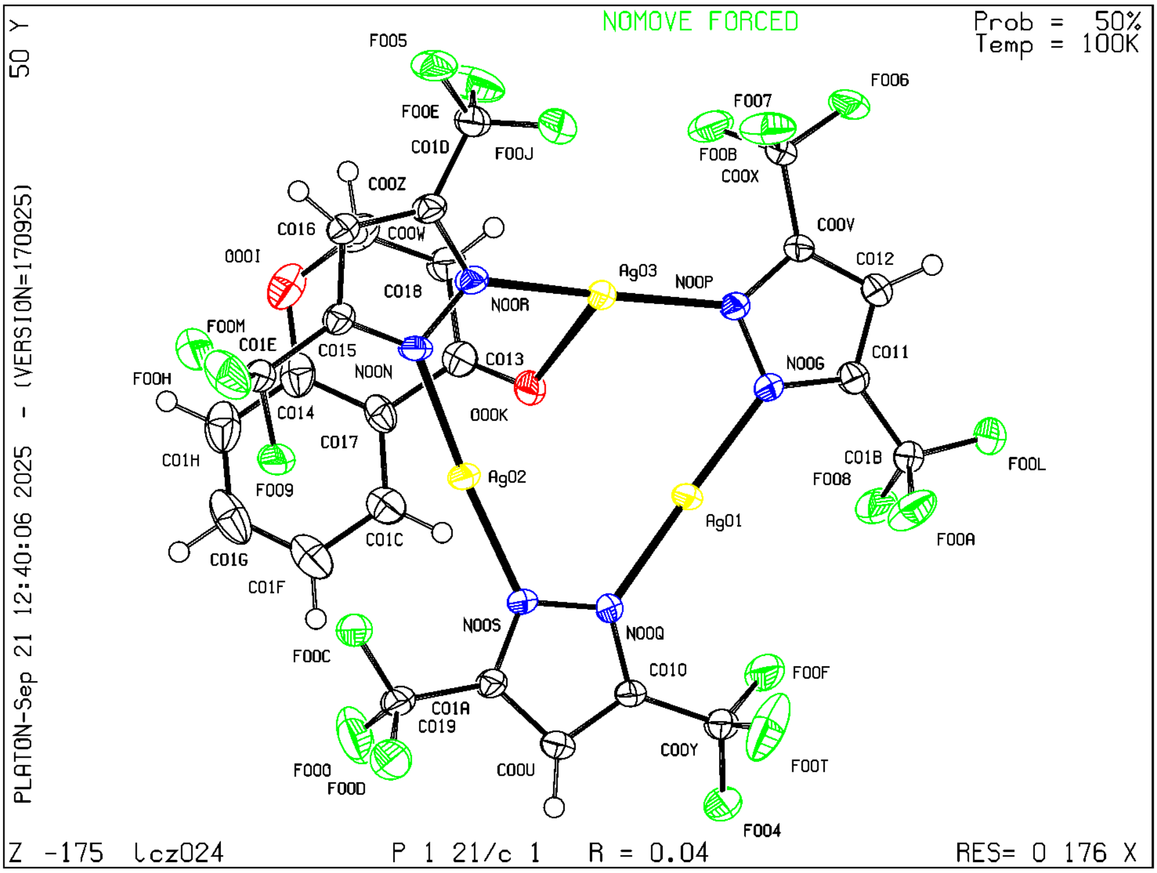

Supplement: Supplementary file 2 — Supporting File 2: anie72462‐sup‐0002‐DataFile.zip. [file ANIE-65-e1218503-s002.zip › supplementary_materials_crystallographic_data submit.zip!/cif_and_checkcif(1-114)/Ag3Pz3_24/Ag3Pz3_24_checkcif.pdf]
